# Case Report: A rarely observed anomaly of the common bile duct—common bile duct duplication

**DOI:** 10.3389/fmed.2025.1584728

**Published:** 2025-07-30

**Authors:** Mehlika Bilgi Kirmaci, Emre Balli, Murat Dayanç, Sezgin Yilmaz

**Affiliations:** Department of General Surgery, Faculty of Medicine, Afyon Health Sciences University, Afyonkarahisar, Türkiye

**Keywords:** duplication of the common bile duct, bile duct, choledoch duplication, surgery, pancreatic cancer

## Abstract

Extrahepatic biliary anomalies are rarely diagnosed. These anomalies are usually detected incidentally after investigating clinical findings in patients such as obstructive jaundice, pancreatitis, or biliary tract malignancies. In our study, we describe common bile duct duplication in a patient scheduled for palliative hepaticojejunostomy due to unresectable pancreatic cancer. We are presenting a case with type I common bile duct anomaly.

## Introduction

Although congenital anomalies of the bile ducts have been reported with a prevalence of 15% in the literature, congenital duplication of the extrahepatic bile ducts is extremely rare. It is characterized by the presence of a septum in the common bile duct or duplication of the common bile duct. The first case of common bile duct anomaly was identified by Vesalius in 1543, and until 1986, 24 cases were reported in total. Additionally, according to a 2002 article published in Japan, 46 cases have been reported since 1968 (1). Bile tract duplications are associated with biliary lithiasis, choledochelelithiasis, cholangitis, pancreatitis, and upper gastrointestinal tract malignancies (2). Before 2007, four types of bile duct duplication were identified. In 2007, Choi et al. found a new type of bile duct duplication, and this type was named type V (3). In this case report, we will present a rare case of common bile duct duplication in a patient diagnosed with inoperable pancreatic cancer.

## Case report

**Figure 1:**
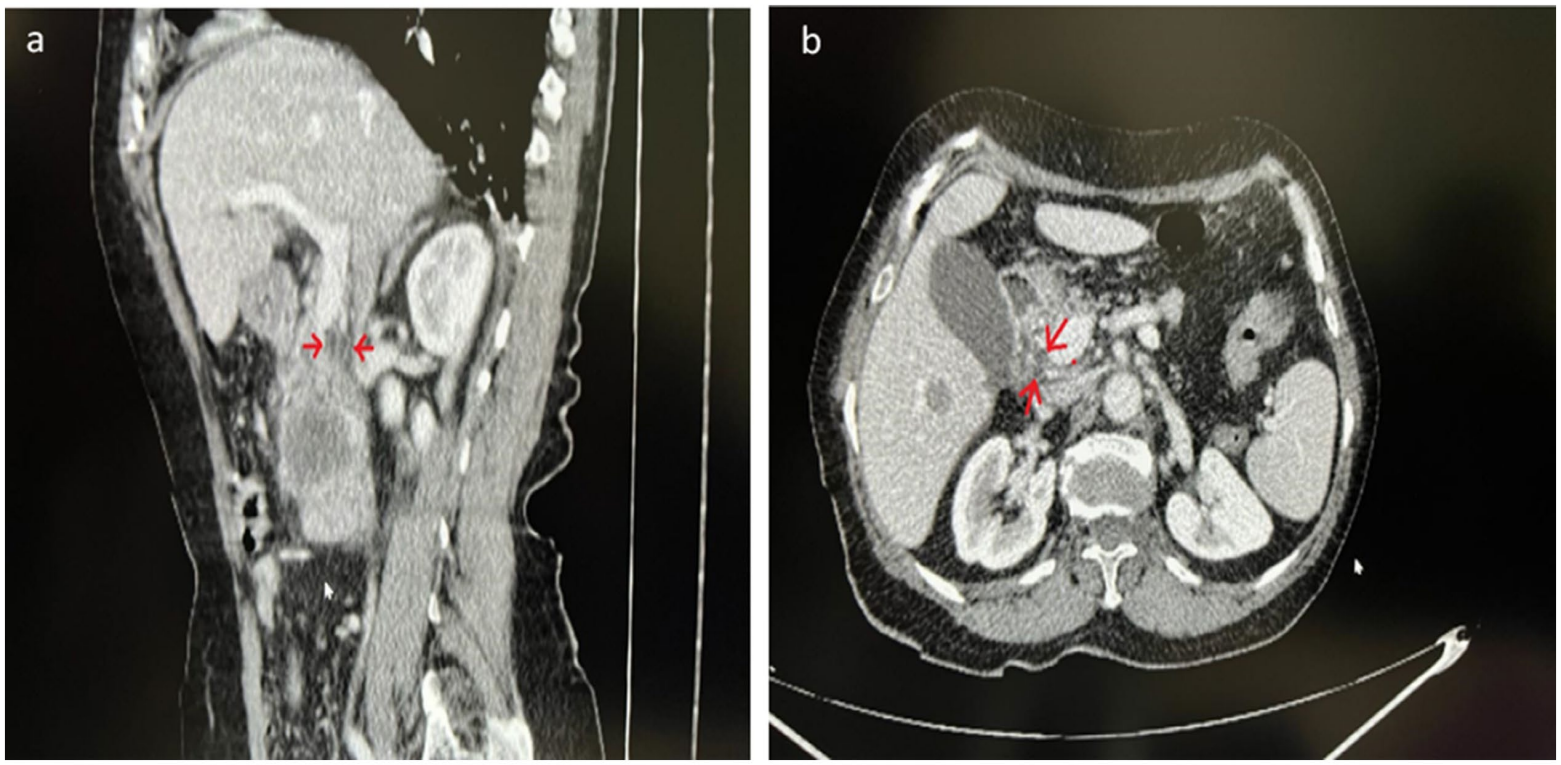
**(a)** Sagittal vision and **(b)** axial vision section of the dynamic CT images shows that the septum within the common bile duct. The septum in the common bile duct is seen in the areas marked with arrows.

A 78-year-old woman was admitted to our hospital with complaints of abdominal pain, nausea, vomiting, and yellowing of the eyes. In the physical examination; high degree Alzheimer’s disease, the scleras were icteric and there was tenderness in the epigastric region. In the laboratory evaluation of the patient, results were obtained as total Bilirubin 10.4 mg/dL, direct bilirubin 9.0 mg/dL ALP 505 iu/ml Gama Glutamil Transferaz (GGT) 488 iu/ml CA19-92500 u/ml. In ultrasonography, a 5×4 cm hypoechoic lesion with irregular contours was observed in the periampullary region. In contrast-enhanced CT, the largest of metastatic lesions 4×5 cm in the liver’s segment 8, and a 4×5 cm mass at the level of the pancreatic uncinate process extending into the duodenum, surrounding the superior mesenteric vein (SMV) more than 180 degrees, were observed. According to clinical and radiological findings, the patient was diagnosed with an inoperable periampullary tumor that invaded the duodenum. ERCP was planned for biliary passage. During the ERCP procedure, an ulcerous mass surrounding tissues was observed in the region compatible with the papilla, which destroyed the papilla and did not allow placement of a stent, and multiple biopsies were taken from the mass. For biliary drainage, it was decided to perform hepaticojejunostomy. During the operation, type I common bile duct duplication was observed (Figure 1). The patient underwent hepaticojejunostomy, gastroenterostomy, and cholecystectomy, and tru-cut biopsies were taken from the periampullary region and liver during the operation. In the post-operative follow-ups, total bilirubin decreased to 2.2 mg/dL and direct bilirubin decreased to 1.5 mg/dL. The patient’s pathology was identified as indifferent pancreatic adenocarcinoma and liver metastasis. At the oncology council, it was decided to continue the patient’s treatment with chemotherapy. The patient, who suffered from Alzheimer’s disease and had an advanced-stage pancreatic tumor, died 3 months after the operation.

## Discussion

Development of the biliary system is a unique process. The liver, gallbladder, and bile duct system arise as a ventral endodermal outgrowth of the hepatic diverticulum, which develops from the distal part of the foregut in the early 4th week of the intrauterine period. While the liver develops from the tissue bud of the ventral foregut diverticulum, intrahepatic bile ducts form from its cranial part, and the extrahepatic bile tree forms from its caudal part. The small caudal part of the hepatic diverticulum forms the gallbladder, and the stalk of the diverticulum forms the cystic duct.

The extrahepatic bile ducts are initially blocked by endoderm-derived epithelial cells called cholangiocytes. Later, the lumen of these channels is formed due to the vacuolation that occurs with the degeneration of these cells. During this process, as the vacuoles merge, the first two parallel ducts are formed, and then they gradually retract, creating the common bile duct. At the end of this process, double bile ducts are seen in the early stages of the intrauterine period, and a common bile duct consisting of a single common hepatic and bile duct is expected to be seen in the postnatal period (3, 4).

Duplication of the extrahepatic bile duct is one of the rarest congenital variants. Mechanisms involved in this developmental anomaly include impairment in the recanalization of the hepatic primordium, random subdivisions of the hepatic diverticulum during the first week of embryogenesis, and early interruption of double common bile duct (DCBD) development. It regresses in early embryogenesis and with normal development (3, 5, 6).

The first description of DCBD was made by Vesalius in 1543. It is a very rare anomaly, with fewer than 30 cases reported in Western studies from 1,543 to 2007. On the other hand, Yamashita et al. reviewed Japanese literature from 1968 to 2002 and found 46 patients with this anomaly (1). The first classification of this anomaly was made by Goor and Ebert (7) and was later modified by Saito et al. (8). Subsequently, the subtypes made by Choi et al. in 2007 are as follows (3, 7, 8).

**Figure 2:**
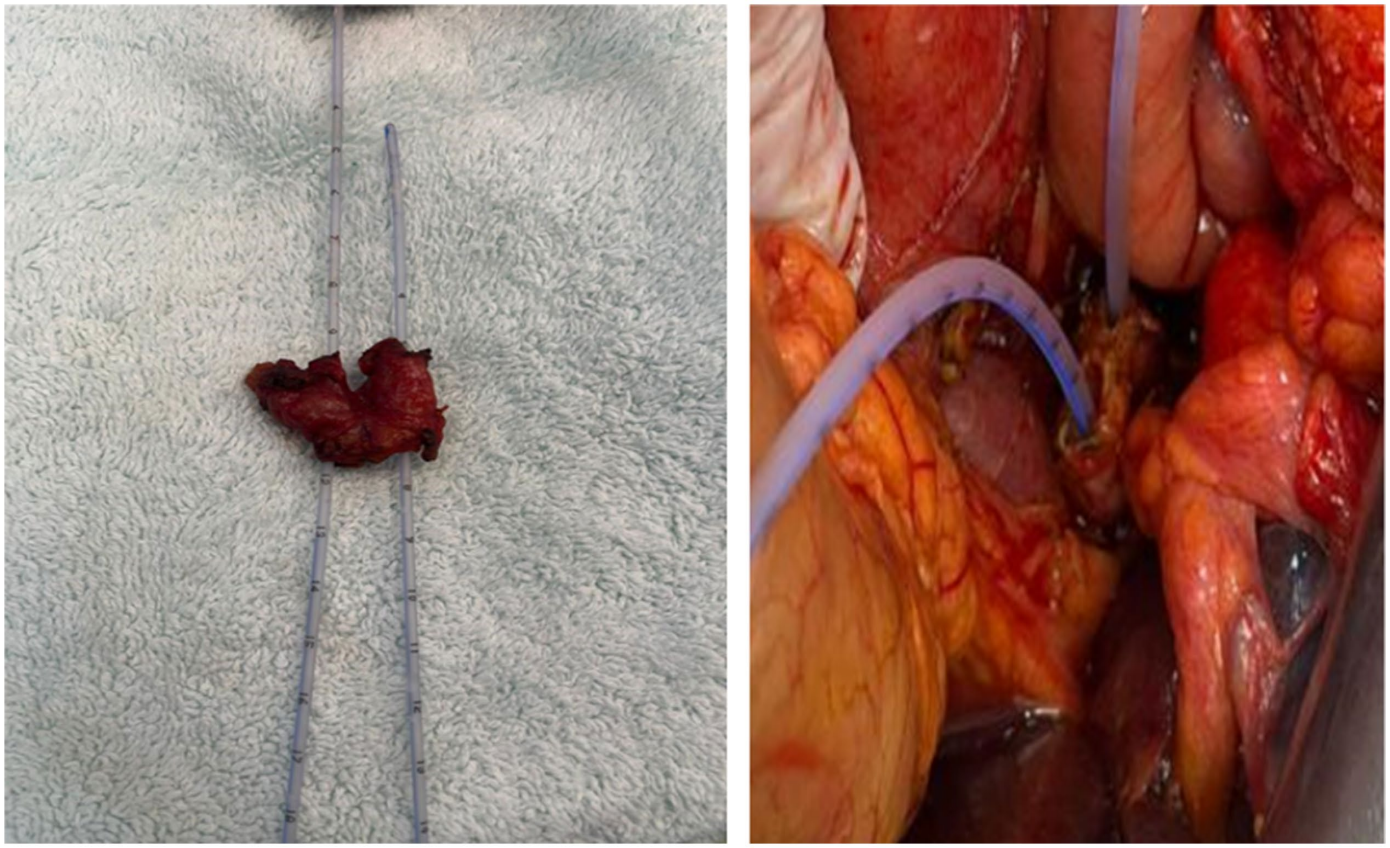
Specimen of the common bile duct with a double lumen. When the common bile duct was cut for hepaticojejunostomy, the catheter was advanced, and the double lumen structure was defined.

The most commonly reported are types III and IV. The least common is type V (9). In our case, we could not visualize a clear biliary tree with ERCP because of the patient’s condition. However, it can be classified as\I variation according to dynamic CT images (Figure 1) and specimen examination (Figure 2).

Variation in the development of the bile duct is a rare anatomical variation that can cause cholangiopathy and further play a role in the etiology of cancer. Chronic inflammation and fibrosis due to bile flow impairment cause the development of malignancy. Obesity, excessive fatty diet, smoking, alcohol, diabetes mellitus, and hyperlipidemia are other factors that cause biliary tract malignancy (1, 3, 10). However, it cannot be detected with a specific tumor marker. A gene analysis that shows these variations, such as colon or breast cancer, cannot be performed. In patients with bile duct anomalies, clinical findings such as recurrent attacks of biliary cholangitis and attacks of pancreatitis are observed. However, in this case, common bile duct variation was observed during palliative treatment for malignancy. When the patient’s medical history was scanned, no previous clinical findings were found (Table 1).

**Table 1:** Classification of the double extrahepatic bile duct.

Type of DCBD	Description	Illustration
Type I	common bile duct with a septum within the lumen	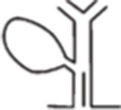
Type II	common bile duct, which bifurcates into two independent drainages (empties separately)	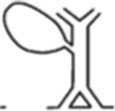
Type IIIa	double biliary drainage without extrahepatic communication channels	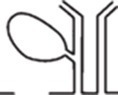
Type IIIb	double biliary drainage without intrahepatic communication channels	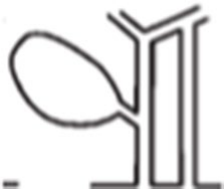
Type IV	double biliary drainage with one or more extrahepatic communicating channels	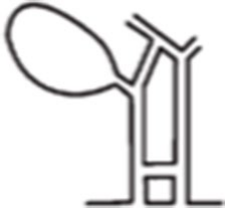
Type Va	single biliary drainage of paired extrahepatic bile ducts without communicating ducts	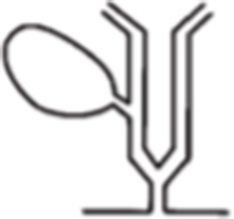
Type Vb	single biliary drainage of paired extrahepatic bile ducts with communicating ducts	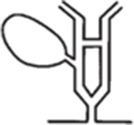

## Conclusion

In conclusion, recurrent episodes of acute pancreatitis and cholangitis due to sludging or obstruction are seen in cases of impaired bile flow, such as common bile duct or pancreaticobiliary junction anomalies. In these cases, a multidisciplinary approach is required for intensive care follow-up, radiological imaging, endoscopic procedures, and surgical treatment according to the clinical findings of the patient. However, in this case, we observed that such anomalies may cause malignant transformation without any clinical findings.

## Data Availability

The raw data supporting the conclusions of this article will be made available by the authors, without undue reservation.
